# mAPKL: R/ Bioconductor package for detecting gene exemplars and revealing their characteristics

**DOI:** 10.1186/s12859-015-0719-5

**Published:** 2015-09-15

**Authors:** Argiris Sakellariou, George Spyrou

**Affiliations:** Center of Systems Biology, Biomedical Research Foundation of the Academy of Athens, 4 Soranou Ephessiou Street, Athens, 115 27 Greece; Department of Informatics and Telecommunications, National & Kapodistrian University of Athens, Athens, Greece

**Keywords:** Feature extraction, Differential expression, Microarray, Gene expression, R, Bioconductor

## Abstract

**Background:**

So far many algorithms have been proposed towards the detection of significant genes in microarray analysis problems. Several of those approaches are freely available as R-packages though their engagement in gene expression analysis by non-bioinformaticians is usually a frustrating task. Besides, only some of those packages offer a complete suite of tools starting from initial data import and ending to analysis report. Here we present an R/Bioconductor package that implements a hybrid gene selection method along with a bunch of functions to facilitate a thorough and convenient gene expression profiling analysis.

**Results:**

mAPKL is an open-source R/Bioconductor package that implements the mAP-KL hybrid gene selection method. The advantage of this method is that selects a small number of gene exemplars while achieving comparable classification results to other well established algorithms on a variety of datasets and dataset sizes. The mAPKL package is accompanied with extra functionalities including (i) solid data import; (ii) data sampling following a user-defined proportion; (iii) preprocessing through several normalization and transformation alternatives; (iv) classification with the aid of SVM and performance evaluation; (v) network analysis of the significant genes (exemplars), including degree of centrality, closeness, betweeness, clustering coefficient as well as the construction of an edge list table; (vi) gene annotation analysis, (vii) pathway analysis and (viii) auto-generated analysis reporting.

**Conclusions:**

Users are able to run a thorough gene expression analysis in a timely manner starting from raw data and concluding to network characteristics of the selected gene exemplars. Detailed instructions and example data are provided in the R package, which is freely available at Bioconductor under the GPL-2 or later license http://www.bioconductor.org/packages/3.1/bioc/html/mAPKL.html.

**Electronic supplementary material:**

The online version of this article (doi:10.1186/s12859-015-0719-5) contains supplementary material, which is available to authorized users.

## Background

The advent of microarray technology gave rise to the development of a plethora of analytical approaches, where each of them try to detect those significant genes among thousands of genes that are enough to summarize all the necessary statistical and biological information for successful classification and pathway analysis. Several of those methods are implemented as open-source R-packages with the anticipation to become broadly engaged by bioscientists in microarray analysis problems. Though, several factors hinder their extensive application particularly from biologists. In some cases the required computational skills while on other the necessity to integrate results of different formats stemming from several packages are some of those factors.

The m*AP*-KL is a hybrid gene selection method, which is based on the hypothesis that among the statistically significant genes in a ranked list, there should be clusters of genes that share similar biological functions related to the investigated disease [1]. The output of this method is a subset of genes consisting of one exemplar per cluster that best describes the phenotypes’ characteristics. The method has been compared against twelve other well-established feature selection methods (eBayes, SAM, RF, BGA, cat, maxT, PLS-CV, ODP, PCA, SNR, *t*-test, HykGene), where all of them applied on a variety of diseases including gene expression data of six neuromuscular diseases and four types of cancer. The overall evaluation results suggest that m*AP*-KL implements an efficient sampling of the ranked gene list, selecting those genes that are necessary for accurate classification while at the same time reflect biological relevance to the respective disease, thus providing a reasonable basis for further biological insights [1].

To provide the research community with the capability to apply m*AP*-KL in any given gene expression dataset, we have implemented this methodology to an open-source R/Bioconductor package accompanied with extra functionalities such as data sampling preprocessing, classification, network analysis, gene annotation analysis, pathway analysis and reporting. The centric idea during the package’s design was to build functions that can either shape an extensive analysis pipeline or used as standalone modules. For instance, a user may import any dataset of raw gene expression data and apply with a single command eight at maximum different preprocessing methods. Then, may analyze any of the preprocessed data with the m*AP*-KL method and conclude to lists of significant genes (exemplars). Classification assessment, annotation analysis, pathway analysis and network characteristics are some of the possible analyses that a user may apply on these exemplars. On the other hand, a user may as well employ any of the available functions to exploit a particular functionality for example, to partition a dataset into train and validation sets, to obtain annotation info for a given list of probe ids, and so on.

In the rest of this article, we will present the key design characteristics that influenced the implementation of this package, and next we will present a thorough workflow highlighted the functional modules of mAPKL. A discussion on other competing packages and how they resemble or differ to mAPKL will follow, and finally the future directions of our package will be outlined.

## Implementation

The mAPKL has been implemented in R as an S4 package that takes advantage of the rich functionality of the ‘ExpressionSet’ (eSet) class [2]. This type of class is designed to accommodate a variety of information including expression data from microarray experiments (assayData), ‘meta-data’ describing samples in the experiment (phenoData), annotations and meta-data about the features on the chip (featureData, annotation), information about the protocol used for processing the samples (protocolData), and a flexible structure to describe the experiment (experimentData). All those different sources of information are handled by class-methods thus the proper manipulation is guaranteed. Besides, using this class objects throughout this package we make feasible any collaboration with other Bioconductor packages hence, extending the meta-analysis options.

From a functional perspective, we have designed and implemented functions that each one of those addresses a particular operation. Indeed, the *loadFiles* function imports gene expression data and convert them into eSet class objects. Hence, this function can be used as a standalone importing and conversion mechanism to eSet class objects by a user who wishes to engage other packages that use those class objects. Similarly, a user may engage the preprocess function to apply at maximum eight different normalization/transformation methods on microarray data and then insert the preprocessed data to another algorithm of statistical inference. In the same way, we may exploit separately each of the available modules. On the other hand, the implemented functions combine a pipeline to assist particularly non-bioinformatician users to run a thorough microarray analysis as we will present in the following section.

## Results

We will present an analysis scenario displaying the functional modules of the mAPKL (see Additional file 1) with the aid of the ‘mAPKLData’ Bioconductor experiment data package (see Additional file 2). During the development of the mAPKL package we also built the mAPKLData package as a necessary supplement to display all different functionalities. It provides the GSE5764 dataset, which is available at the NCBI Gene Expression Omnibus and includes gene expression data from a breast cancer study published by Turashvili et al. [3] that contains 30 samples related to breast cancer (20 normal and 10 tumor samples), based on Affymetrix HG-U133_Plus_2 microarray platform.

### Data import and manipulation

We developed the *loadFiles* function that takes as input text tab-delimited files and converts them into eSet class objects. Typically, a gene expression analysis requires a pair of files for training and optionally another pair for validation purposes. Each pair includes a file with the gene expressions in a matrix format, where rows represent probe ids and columns represent samples, and a file with the relevant class labels. In our case, the class label file should have a minimum of two columns with headers, for example ‘title’ and ‘type’. In the ‘type’ column we assign the class labels of the samples where ‘0’ stands for control and ‘1’ stands for ‘treatment’ samples. The *loadFiles* function, supports importing and converting both, training and validation files, in a single command line.

A further data manipulation attribute offered in the mAPKL package is the dataset partition into train and validation sets according to a user defined percentage. In particular, the *sampling* function takes as input an eSet class objects describing a cohort of samples and split into separate train and test sets following a user’s predefined percentage. The minimum percentage of samples that might be kept for validation purposes is 10 % and the user is always confident that the function creates stratified sets in relation to their class analogy within the initial dataset. Moreover, the samples’ selection follows a random pick based on a pre-defined seed number, which on the one hand guarantees reproducibility while on the other eliminate any bias during the selection. In the current scenario, we loaded the mAPKLData, and then with the aid of the sampling function we kept 40 % of the samples for validation purposes, which produced a train set with twelve normal and six tumor samples, and a validation set with eight normal and four disease samples. Those scenario steps require the following R commands.

- library(mAPKL)
- library(mAPKLData)
- data(mAPKLData)
- breast < − sampling(Data = mAPKLData, valPercent = 40, classLabels = “type”, seed = 135)

### Data normalization and transformation

**Fig. 1:**
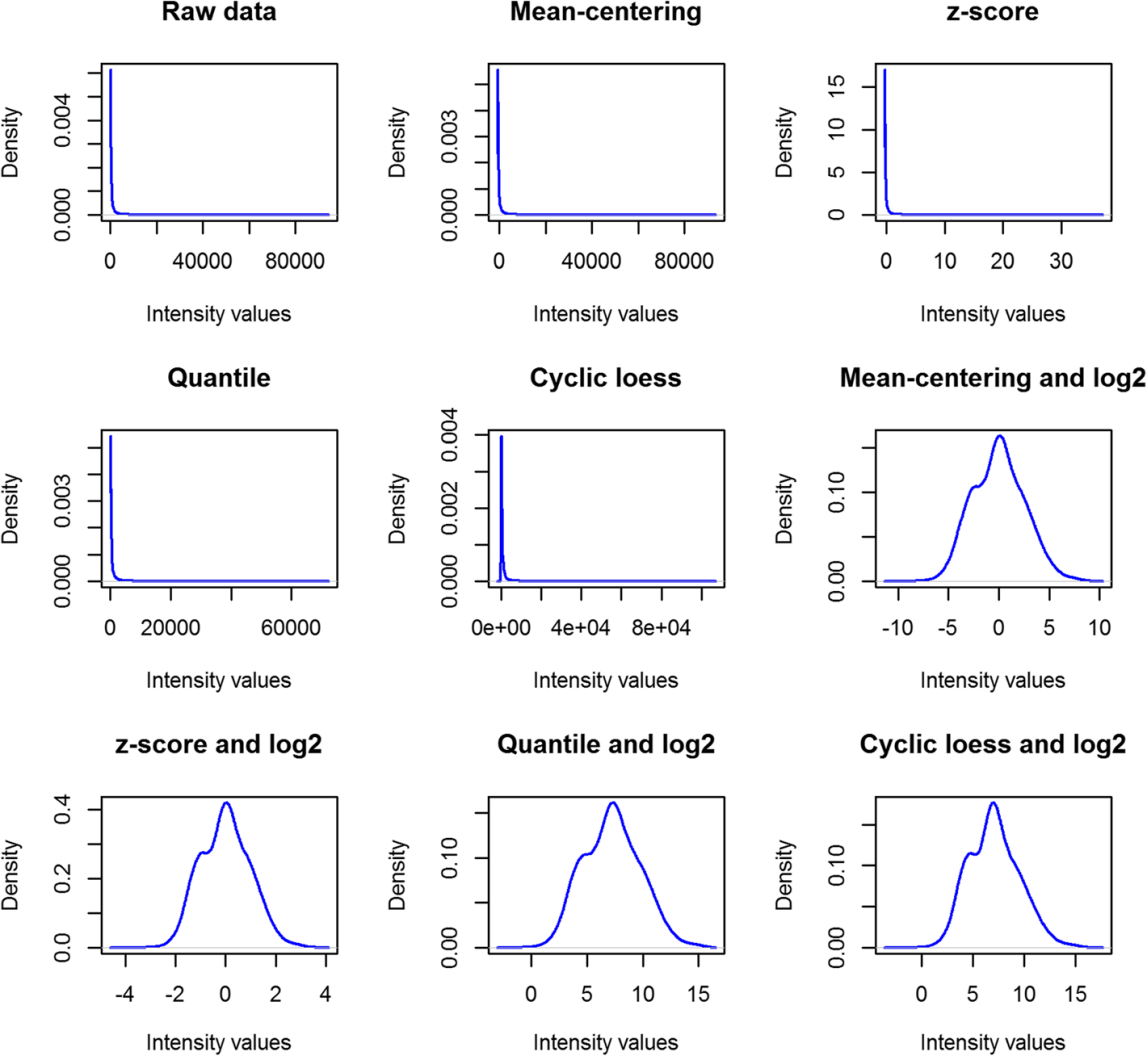
Density plots of normalized intensity values

The package accommodates a practical preprocessing functional unit that supports log2 transformation along with four different normalization methods including mean-centering, z-score, quantile and cyclic loess. In particular, the *preprocess* function produces an S3 class object, i.e. a list with maximum nine available options, Table 1, as well as a multi-graph image with the density plots of the preprocessed data for each method, Fig. 1. Hence, the user may proceed with a gene selection analysis for any of the preprocessed gene expression values. In the following section, we run eight different scenarios and assess their classification results before reaching to a decision about the most appropriate normalization scheme for the mAPKLData. The following two lines of code apply the described normalization\transformation options instantly to the train and test sets respectively.

**Table 1:** The available options in the preprocessing functional unit

Abbreviation	Description
rawdata	The initial gene expression values
mc	The values after ‘mean-centering’ normalization
z	The values after ‘z-score’ normalization
q	The values after ‘quantile’ normalization
cl	The values after ‘cyclic loess’ normalization
mcL2	The values after log2 transformation and ‘mean-centering’ normalization
zL2	The values after log2 transformation and ‘z-score’ normalization
qL2	The values after log2 transformation and ‘quantile’ normalization
clL2	The values after log2 transformation and ‘cyclic loess’ normalization

- normTrainData < − preprocess(breast$trainData)
- normTestData < − preprocess(breast$testData)

### Gene selection analysis

The core function of this package is the *mAPKL* that implements the hybrid feature selection methodology. It takes as input an eSet class object with the training data and several predefined parameters necessary for the intrinsic statistical analysis and clustering. It may also accept a validation eSet object to directly apply on it the results of the m*AP*-KL analysis. This function returns an object of ‘mAPKLRes’ S4 class where its slots embody the matrix of the top *N* ranked genes, the clusters and their respective exemplars, the training and validation eSet objects of the exemplars, along with statistical information such as p-value, adjusted p-value and fold-change for all genes. In relation to the gene expression analysis of the mAPKLData, we carried out eight different m*AP*-KL analyses and concluded to eight different subsets of exemplars, which will be assessed for their discrimination power. In the next lines of code, we employ the gene expression values resulted from the cyclic loess normalization on log2 transformed data, and then apply the m*AP*-KL method. Similarly we may engage any preprocessed set of gene expression values and ask for their exemplars.

- exprs(breast$trainData) < − normTrainData$clL2.normdata
- exprs(breast$testData) < − normTestData$clL2.normdata
- out.clL2 < − mAPKL(trObj = breast$trainData, classLabels = “type”,
- valObj = breast$testData, dataType = 7)

### Classification

The following functional module provides classification estimates for the selected genes. In particular, it utilizes the exemplars’ eSet objects from the ‘mAPKLRes’ class to run an SVM based cross-validation classification test to quantify the discrimination power of the gene exemplars. The necessary parameters for running the SVM classifier are computed dynamically with the *tune.svm* function of the ‘e1071’ R-package [4]. The classification measures are calculated through a mAPKL’s function called *metrics* and include the Area Under the Curve (AUC), the Matthews correlation coefficient (MCC), the accuracy (ACC), the true negative rate (TNR) or specificity and the true positive rate (TPR) or sensitivity. This function performs classification through the SVM algorithm and produces a classification result either on the training set or on a validation set. During this analysis we assessed the performance on the validation set using the following SVM parameters: ‘linear’ kernel and 5-folds cross-validation (although other options are feasible) and the R command is structured as follows:

- clasPred < − classification(out.clL2@exemplTrain, “type”, out.clL2@exemplTest).

According to the classification results, Table 2, the exemplars’ list produced after log2 transformation and cyclic loess normalization achieved the best discrimination results and will be further explored from a pathway and a network-topology perspectives.

**Table 2:** Classification performance of gene exemplars per preprocessing method

Method	Exemplars	AUC	MCC	ACC	TNR	TPR
clL2	15	0.94	0.84	92.0	0.88	1.00
mcL2	40	0.88	0.82	92.0	1.00	0.75
qL2	40	0.88	0.82	92.0	1.00	0.75
z	17	0.81	0.62	83.0	0.88	0.75
mc	28	0.81	0.62	83.0	0.88	0.75
cl	17	0.75	0.63	83.0	1.00	0.50
q	14	0.69	0.41	75.0	0.88	0.50
zL2	39	0.62	0.43	75.0	1.00	0.25

### Gene annotation and pathway analysis

The next functional module exploits the microarray chip annotation file, when available, to collect necessary genome oriented information so as to facilitate other types of genome analysis such as pathway analysis. The ‘Annot’ S4 class provides slots for gene ‘symbol’, ‘entrezId’, ‘ensembleId’ and chromosomal location info of the exemplars. Thus, the user not only has at hand a valid conversion mechanism between probes and genes but also several additional meta-data for other types of analysis like pathway or Gene Ontology. For this purpose we run as follows the *annotate* function with the argument ‘chip’ equal to ‘hgu133plus2.db’ since this is the relevant microarray chip platform for the mAPKLData dataset.

- gene.info < − annotate(out.clL2@exemplars,“hgu133plus2.db”)

**Fig. 2:**
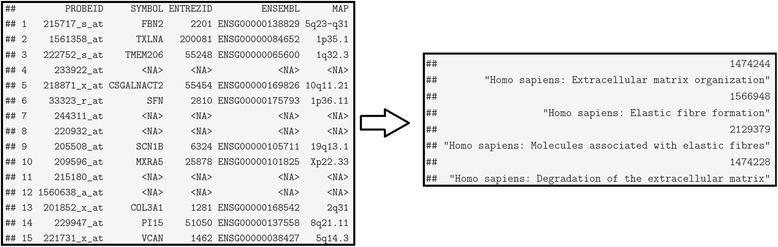
From annotation to pathways. The 15 exemplars (probe ids) match to ten different ‘entrez ids’, which in turn are found to be related to four pathways

Then, we may easily exploit the ‘Annot’ class object produced by the *annotate* function to perform a pathway analysis utilizing the ‘Reactome’ pathway database [5] through the *probes2pathways* function. Particularly, we first remove the ‘NA’ entries and then map the ‘ENTREZID’ to the ‘Reactome’ pathway identifiers, Fig. 2. Thus, for any given set of exemplars a user may effortlessly identify the relevant pathways.

### Network characteristics

**Fig. 3:**
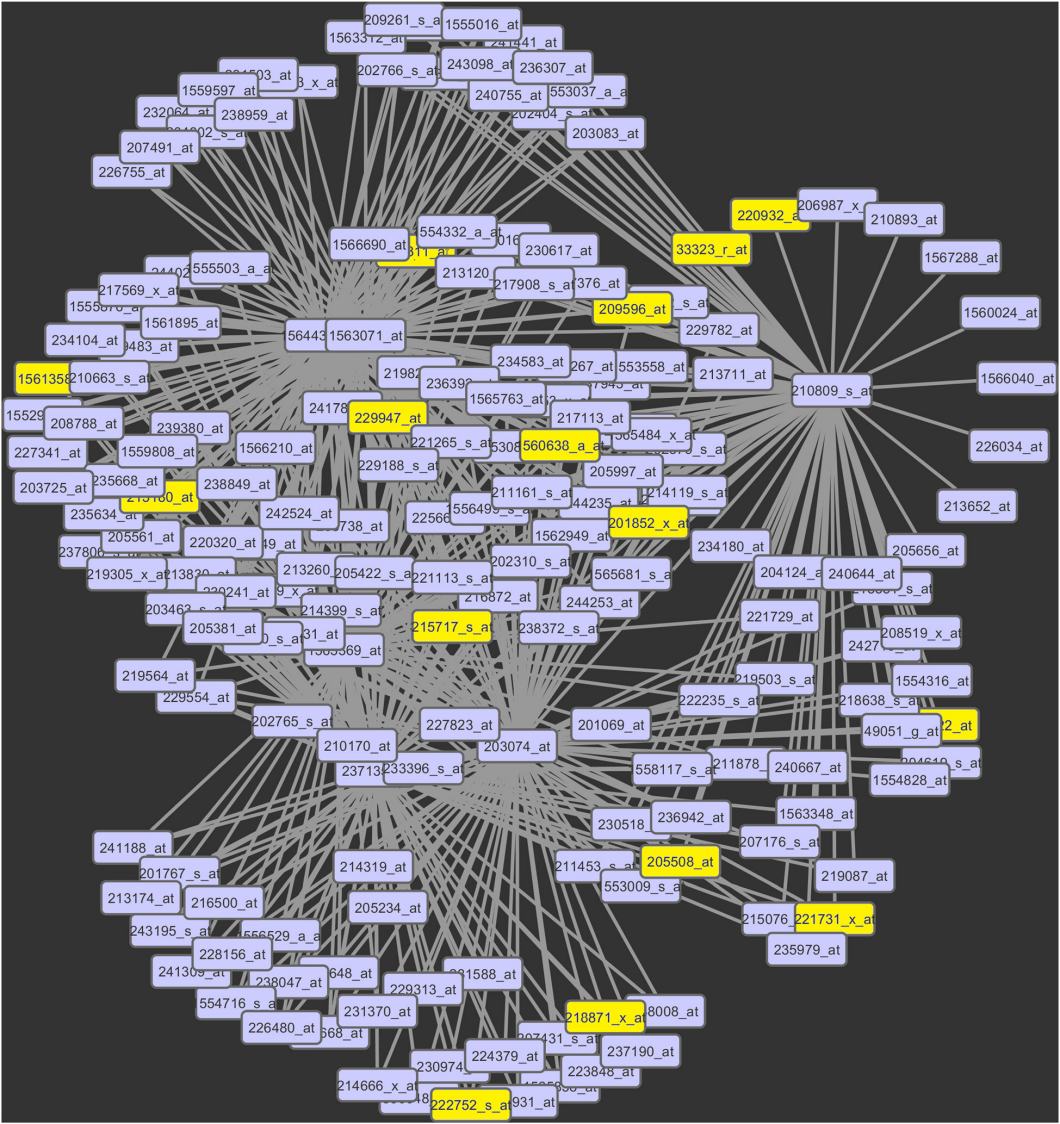
A network graph of the weighted local degree of centrality

The *netwAttr* function deals with the network characteristics of the top *N* ranked genes but more importantly of the gene exemplars. Three different types of centralities (degree, closeness, betweenness) and a measure for clustering coefficient, called transitivity, are estimated with this function. The degree centrality of a node refers to the number of connections or edges of that node to other nodes. The closeness centrality describes the reciprocal accumulated shortest length distance from a node to all other connected nodes. The betweeness centrality depicts the number of times a node intervenes along the shortest path of two other nodes. Transitivity measures the degree of nodes to create clusters within a network. For all four network measures we provide both global and local values. Moreover, the *netwAttr* provides a weighted edge list (Node1-Node2-weight) based on the top *N* ranked genes, as a front end to network and graph packages for further analysis and visualization. Particularly, the *netwAttr* function computes the network degree, closeness, betweenness and transitivity. We compose an edge list (Node1-Node2-weight) based on the top *N* ranked genes (200 in this example) to interface with other network related R-packages like igraph [6] or with software tools outside the R platform like Cytoscape [7]. Indeed, we plot a network graph to present the relations-connections among the top *N* ranked genes, Fig. 3, through Cytoscape. For both significance and illustration purposes we display only the nodes with local weighted degree greater than the global weighted degree plus 2 times the standard deviation. The calculations of those network characteristics are based on the “clr” [8] network reconstruction method. However, there are two more available options, including the “aracne.a” and “aracne.m’ [9] algorithms. The basic syntax for this function is the following, although some additional R commands are necessary to create the described output values for the graph. Details are included in the mAPKL’s vignette, net.attr < − netwAttr(out.clL2).

### Report generation

With regard to the *report* function, the user may produce a summarized report in html format that presents the results in all different stages of analysis. Specifically, the first section depicts the samples' names and their respective class labels, the second section presents the exemplars along with their statistical results and network characteristics, the third section deals with the classification results and illustrates the performance metrics achieved in either cross-validation or hold-out validation. Finally, the fourth section of this report cites the annotation info per exemplar relevant to the chip technology.

## Discussion

### Comparison with other microarray data analysis software

There are several software tools related to differential gene expression and samples classification. All of those tools can be roughly classified based on two criteria; the software technology used and the scope of the analysis they offer. Regarding the first criterion, software applications can be further divided into Web applications, desktop java applications and R-packages. Concerning the second criterion, there are methods for gene selection, for classification of samples, and methods that engage clustering either for gene selection or for unknown samples categorization. mAPKL package is the implementation of a hybrid gene selection algorithm under the R/Bioconductor framework, which also includes additional functionalities, aiming at providing a thorough gene expression analysis pipeline. In this context, we compared the mAPKL package against the R implementations of the feature selection methods included in the previously published m*AP*-KL evaluation [1] as well as against other R-packages that offer functionalities related to data sampling, gene selection and classification.

Regarding the R-packages included in the following comparisons, there are seven feature selection and five classification software tools. Particularly the feature selection packages represent the following feature selection approaches: the ‘edge’ [10] package implements the Optimal Discovery Procedure (ODP) [11] feature selection method, the ‘limma’ [12] includes the eBayes [13] approach, the ‘multtest’ [14] provides the maxT [15] procedure, the ‘plsgenomics’ [16] contain the Partial Least Squares (PLS) [17] method, the ‘randomForest’ [18] implements the RF [19] algorithm, the ‘samr’ [20] carries out the Significance Analysis of Microarrays (SAM) [21] method, and the ‘st’ [22] package implements the cat score [23]. As far as the classification packages that most resemble to mAPKL are the ‘caret’ [24], the ‘ClassifyR’ [25], the ‘CMA’ [26], the ‘MCRestimate’ [27], and the ‘MLInterfaces’ [28].

Firstly, the packages have been compared in terms of several functional criteria, Table 3, including ‘Data’ manipulation, ‘Significance analysis’, ‘Classification’, ‘Annotation analysis’, computation of ‘Network characteristics’ and ‘Reporting’. In addition, we have included subcategories in some of those criteria so as to provide an in-depth comparison. Thus, the ‘Data’ category falls into four subcategories related to functions for data importing, normalization and transformation, dataset sampling, and eSet class utilization. Regarding the ‘Classification’ criterion we have distinguished the prediction results from the available performance metrics.

**Table 3:** Comparison of features between mAPKL and other related R-packages

Package	Data				Significance analysis	Classification		Annot. analysis	Network chars	Reporting
	Import	Use eSet	Norm.	Sampling		Pred.	Perf. metrics			
mAPKL	Yes	Yes	Yes	Yes	Yes	Yes	Yes	Yes	Yes	Yes
edge	No	Yes	Yes	No	Yes	No	No	No	No	No
limma	Yes	No	Yes	No	Yes	No	No	Yes	No	No
multtest	No	Yes	No	No	Yes	No	No	No	No	No
plsgenomics	No	No	Yes	No	Yes	Yes	No	No	No	No
randomForest	No	No	No	No	Yes	Yes	No	No	No	No
samr	No	No	Yes	No	Yes	No	No	No	No	No
st	No	No	No	No	Yes	No	No	No	No	No
caret	No	No	No	Yes	Yes	Yes	Yes	No	No	No
ClassifyR	No	Yes	No	No	Yes	Yes	Yes	No	No	No
CMA	No	Yes	No	Yes	Yes	Yes	Yes	No	No	No
MCRestimate	No	Yes	No	No	Yes	Yes	Yes	No	No	No
MLInterfaces	No	Yes	No	Yes	Yes	Yes	Yes	No	No	No

Secondly, we employed the mAPKLData package to compare the classification performance of mAPKL against the other seven feature selection packages. For equality purposes we engaged the same preprocessing approach (the cyclic loess normalization on log2 transformed data), and we decided to keep subsets of genes of the same length based on the 15 “exemplars” of the mAPKL. Besides, we utilized the *classification* function included in our package to evaluate the top-15 gene subsets from the other seven approaches, and the classification results are presented in Table 4.

**Table 4:** Classification performance among feature selection methods for a subset of 15 top-ranked genes (probe ids)

Method	AUC	MCC	ACC	TNR	TPR
m*AP*-KL	0.94	0.84	92.0	0.88	1.00
cat	0.88	0.82	92.0	1.00	0.75
ODP	0.88	0.82	92.0	1.00	0.75
RF	0.81	0.62	83.0	0.88	0.75
maxT	0.75	0.63	83.0	1.00	0.50
PLS	0.69	0.41	75.0	0.88	0.50
eBayes	0.62	0.43	75.0	1.00	0.25
SAM	0.62	0.43	75.0	1.00	0.25

The accumulated comparison results suggest that the mAPKL is more than an R implementation of an effective feature selection method or a classification package. It is a complete and comprehensive gene expression analysis package offering a set of functions to assist the genomics researchers to perform a rigorous analysis starting from raw gene expression data and ending to annotated significant genes accompanied with certain evaluation metrics and networking properties, assembling the information in an auto-generated analysis report.

As part of the Bioconductor project, the mAPKL package is freely available under the GPL-2 or later license accompanied with detailed help pages per class and function. Besides, an elaborate vignette introduces all available functionalities through a case study scenario that is based on the ‘mAPKLData’ Bioconductor experiment data package. Thus, the user can see both illustrated codes and executed outputs to become easily accustomed to the package. Moreover, the Bioconductor project guarantees the easy implementation and platform independence, the versioning of the forthcoming package releases and the obliged that the package will be maintained by the author, which includes response to bug reports or queries from other users as well as checking periodically the functionality of the package.

### Future directions

In relation to the potential expansions of the mAPKL package, we intend to include alternative methods for gene selection as well methods related to functional enrichment analysis.

## Conclusions

We have presented an R/Bioconductor package named mAPKL concerning the implementation of the m*AP*-KL method for efficient gene selection from gene expression data. Additionally, it incorporates several features including data sampling to create train and validation sets, various preprocessing methods, functions for measuring the classification performance of the selected genes and gene annotation analysis to facilitate other types of analysis like pathway analysis. Moreover, it constructs networks based on the top *N* genes of our methodology and exploits several network characteristics of the ‘exemplars’ to produce graphical representations of the cellular network topology. In general, it is a user friendly R/Bioconductor package for gene expression data analysis that includes a comprehensive suite of functions. Those functions include default values to facilitate users without advanced computational or statistical background and collaborate smoothly to integrate into a custom analysis pipeline.

## Availability and requirements

**Project name: **mAPKL

**Project home page: **http://www.bioconductor.org/packages/3.1/bioc/html/mAPKL.html

**Operating system(s): **Platform independent

**Programming language: **R

**Other requirements:**R (> = 3.2.0), Biobase

**License:**GPL (> = 2)

**Any restrictions to use by non-academics: **None

## Additional files

Additional file 1:
**Package ‘mAPKL_1.1.1.tar.gz’ including source code, documentation.** (GZ 1979 kb) Additional file 2:
**Package ‘mAPKLData_1.0.0.tar.gz’ including microarray data and documentation.** (GZ 9281 kb)
