# Supplementary material for: mAPKL: R/ Bioconductor package for detecting gene exemplars and revealing their characteristics
Source: BMC Bioinformatics. 2015 Sep 15;16(1):291. doi: 10.1186/s12859-015-0719-5 (PMC4572678; doi:10.1186/s12859-015-0719-5)
Supplement: Additional file 1: — Package ‘mAPKL_1.1.1.tar.gz’ including source code, documentation. (GZ 1979 kb) [file 12859_2015_719_MOESM1_ESM.gz › mAPKL/inst/doc/mAPKL.pdf]

Bioconductor mAPKL Package

Argiris Sakellariou[1,2]

[1] Biomedical Informatics Unit, Biomedical Research Foundation of the Academy of Athens, Athens, Greece
[2] Department of Informatics and Telecommunications, National and Kapodistrian Univ. of Athens, Athens, Greece
argisake@gmail.com

July 21, 2015

Contents

1 Introduction 1

2 Identification of deferentially expressed genes 2
- 2.1 Breast cancer data . . . 2
- 2.2 Data normalization and transformation . . . 3
- 2.3 mAPKL gene selection . . . 3
- 2.4 Building and evaluating classification models . . . 5

3 Advanced usage of the package 6
- 3.1 Annotation analysis . . . 6
- 3.2 Network characteristics . . . 7

4 Reporting 10

5 Session info 10

6 Reference 11

1 Introduction

The mAPKL bioconductor R package implements a hybrid gene selection method, which is based on the hypothesis that among the statistically significant genes in a ranked list, there should be clusters of

genes that share similar biological functions related to the investigated disease.Thus, instead of keeping a number of N top ranked genes, it would be more appropriate to define and keep a number of gene cluster exemplars.

The proposed methodology combines filtering and cluster analysis to select a small yet highly discriminatory set of non-redundant genes. Regarding the filtering step, a multiple hypothesis testing approach from *multtest* r-package (maxT) is employed to rank the genes of the training set according to their differential expression.Then the top N genes (e.g. N = 200) are reserved for cluster analysis. First the index of Krzanowski and Lai as included in the *ClusterSim* r-package is applied on the disease samples of the training set to determine the number of clusters. The Krzanowski and Lai index is defined by $DIFF(k) = (k-1)^{\frac{2}{p}} W_{k-1} - k^{\frac{2}{p}} W_k$ when choosing the number of clusters (k) to maximize the quantity $KL(k) = \left| \frac{DIFF(k)}{DIFF_(k+1)} \right|$. The W_k denotes the within-cluster sum of squared errors.

Finally, cluster analysis is performed with the aid of Affinity Propagation (AP) clustering algorithm, which detects $n(n = k$ the Krzanowski and Lai index) clusters among the top N genes, the so called exemplars.Those n exemplars are expected to form a classifier that shall discriminate between normal and disease samples (Sakellariou et al. 2012, *BMC Bioinformatics* **13**:270). This package implements the pre-mentioned methodology through a core function, the *mAPKL*. In the upcoming sections follows a guidance of the included functions and its functionality through differential expression analysis scenarios on a breast cancer dataset (GSE5764) which is part of the *mAPKLData* package.

2 Identification of deferentially expressed genes

2.1 Breast cancer data

Throught this tutorial we utilized a publicly available breast cancer dataset comprised of 30 samples, where 20 of them represent normal cases and the remaining 10 samples stand for tumor cases. We first load the package and then the breast cancer data. Then with the aid of the *sampling* function we create a separate training and validation sets where 60% of the samples will be used for training and the rest 40% of the samples will be used for evaluation purposes.

```
library(mAPKL)
library(mAPKLData)
data(mAPKLData)
varLabels(mAPKLData)
breast <- sampling(Data=mAPKLData, valPercent=40, classLabels="type", seed=135)
```

The *sampling* function returns an S3 class (breast) with two eSet class objects that nest the relevant training and validation sampes. Those two objects are used throughout the rest of the analysis.

```
breast

## $trainData
```

```
## ExpressionSet (storageMode: lockedEnvironment)
## assayData: 54675 features, 18 samples
##   element names: exprs
## protocolData: none
## phenoData
##   sampleNames: GSM134584 GSM134690 ... GSM134695 (18 total)
##   varLabels: title type
##   varMetadata: labelDescription
## featureData: none
## experimentData: use 'experimentData(object)'
##   pubMedIds: 17389037
## Annotation: hgu133plus2
##
## $testData
## ExpressionSet (storageMode: lockedEnvironment)
## assayData: 54675 features, 12 samples
##   element names: exprs
## protocolData: none
## phenoData
##   sampleNames: GSM134691 GSM134588 ... GSM134692 (12 total)
##   varLabels: title type
##   varMetadata: labelDescription
## featureData: none
## experimentData: use 'experimentData(object)'
##   pubMedIds: 17389037
## Annotation: hgu133plus2
```

2.2 Data normalization and transformation

We perform normalization to the expression values through the *preprocess* function.

```
normTrainData <- preprocess(breast$trainData)
normTestData <- preprocess(breast$testData)
```

The *preprocess* function produces a list of several available normalization and transformation options. Besides density plots per method are produced and saved to current working directory to assist the user to decide upon which method to select before proceeding to mAPKL analysis.

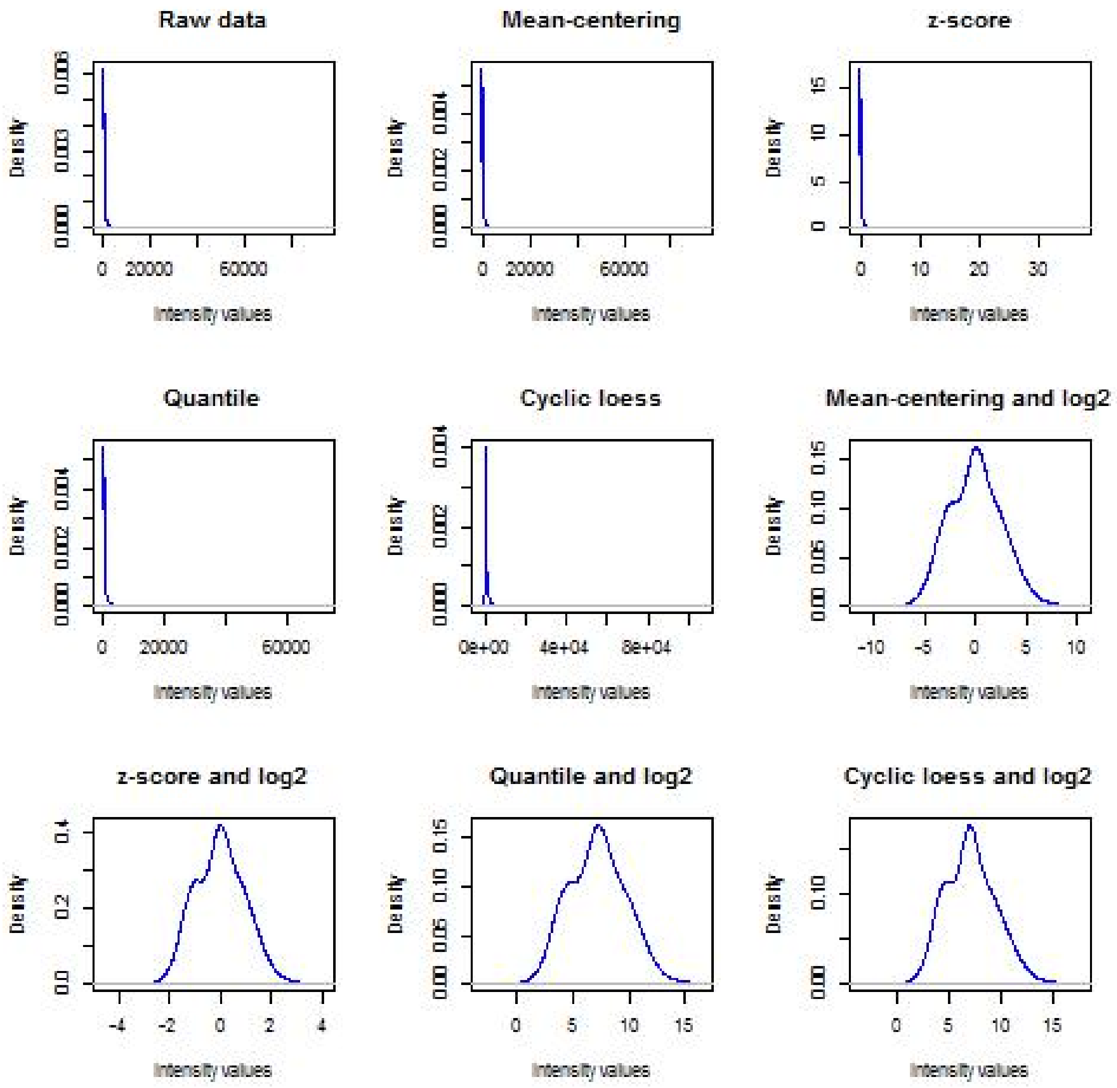

Figure 1: Density plots of normalized intensity values

```
attributes(normTrainData)

## $names
## [1] "rawdata"       "mc.normdata"   "z.normdata"    "q.normdata"    "cl.normdata"
## [6] "mcL2.normdata" "zL2.normdata"  "qL2.normdata"  "clL2.normdata"
```

The following graph presents the density plots of 8 possible normalization process with or without log2 transformation. The *preprocess* function applies all of them and it is up to the user, which one will engage for the rest of the analysis. In brief, the available approaches are mean-centering, z-score, quantile, and cyclic loess. During this case study we will proceed with the expression values folowing log2 transformation and cyclic loess normalization.

2.3 mAPKL gene selection

In this example we employ the expresion values of log2 transformation and cyclic loess normalization to proceed with the *mAPKL* analysis.

```
exprs(breast$trainData) <- normTrainData$clL2.normdata
exprs(breast$testData) <- normTestData$clL2.normdata
out.clL2 <- mAPKL(trObj = breast$trainData, classLabels = "type",
    valObj = breast$testData, dataType = 7)

## b=10 b=20 b=30 b=40 b=50 b=60 b=70 b=80 b=90 b=100
## b=110 b=120 b=130 b=140 b=150 b=160 b=170 b=180 b=190 b=200
## b=210 b=220 b=230 b=240 b=250 b=260 b=270 b=280 b=290 b=300
## b=310 b=320 b=330 b=340 b=350 b=360 b=370 b=380 b=390 b=400
## b=410 b=420 b=430 b=440 b=450 b=460 b=470 b=480 b=490 b=500
## b=510 b=520 b=530 b=540 b=550 b=560 b=570 b=580 b=590 b=600
## b=610 b=620 b=630 b=640 b=650 b=660 b=670 b=680 b=690 b=700
## b=710 b=720 b=730 b=740 b=750 b=760 b=770 b=780 b=790 b=800
## b=810 b=820 b=830 b=840 b=850 b=860 b=870 b=880 b=890 b=900
## b=910 b=920 b=930 b=940 b=950 b=960 b=970 b=980 b=990 b=1000

## Please wait!  The (KL) cluster indexing may take several minutes...
## Asking for 15 number of clusters
## fc according to limma
```

2.4 Building and evaluating classification models

After having get the exemplars from *mAPKL* analysis we build an SVM classifier to test their discriminatory performance. Regarding the SVM setup, we utilize a linear kernel for which the cost attribute is infered by the tune.svm function. however, the user may freely use another kernel and a different Cross Validation approach than 5-folds.

```
clasPred <- classification(out.clL2@exemplTrain, "type", out.clL2@exemplTest)

## The training set has 12 Negative and 6 Positive samples.  Using k-fold=5 C-V
## ############ THE BEST PARAMETERS TUNING STAGE ##################
## ############# THE TRAINING STAGE ########################

##
## Call:
## svm.default(x = train.mtx, y = lbls, scale = FALSE, type = "C-classification",
##     kernel = "linear", gamma = best_gamma, cost = best_cost, cross = k_fold)
##
```

```
##
## Parameters:
##    SVM-Type:  C-classification
##  SVM-Kernel:  linear
##        cost:  2
##       gamma:  0.125
##
## Number of Support Vectors:  5
## ############# THE PREDICTION STAGE #####################
##           Test Labels Prediction Labels
## GSM134691           0                 0
## GSM134588           0                 0
## GSM134688           0                 0
## GSM134694           0                 1
## GSM134697           0                 0
## GSM134700           0                 0
## GSM134687           0                 0
## GSM134709           0                 0
## GSM134710           1                 1
## GSM134698           1                 1
## GSM134689           1                 1
## GSM134692           1                 1
## Negative samples:  8
## Positive samples:  4
## TN=7
## FP=1
## TP=4
## FN=0
## AUC=0.94
## Accuracy=92.00
## MCC=0.84
## Specificity=0.88
## Sensitivity=1.00
```

The output of the *classification* inform us about the SVM set up, the number of Support Vectors and finally show the the predicted labels along with the initial. In this example there is a validation set different from the training set and therefore we may use the respective labels to obtain the performance characteristcs. The relevant function *metrics* called inside the *classification* function, calculates five key meassures: the Area Under the ROC curve AUC, the classification accuracy, the Matthews correlation

coefficient MCC classification meassure, the degree of true negative's identification Specificity, and finally the degree of true positive's identification Sensitivity.

3 Advanced usage of the package

3.1 Annotation analysis

For each contemporary chip technology, there is a relevant annotation file, in which the the user may drag several *genome oriented* information. Regarding the breast cancer microarray data, the gene expression values were stored on Affumetrix gene chips. Using the *annotate* function, the user may obtain several info related to probe id, gene symbol, Entrez id, ensembl id, and chromosomal location.

```
gene.info <- annotate(out.clL2@exemplars, "hgu133plus2.db")
gene.info@results

##          PROBEID     SYMBOL ENTREZID         ENSEMBL      MAP
## 1   215717_s_at       FBN2     2201 ENSG00000138829 5q23-q31
## 2    1561358_at      TXLNA   200081 ENSG00000084652   1p35.1
## 3   222752_s_at    TMEM206    55248 ENSG00000065600   1q32.3
## 4     233922_at       <NA>     <NA>            <NA>     <NA>
## 5   218871_x_at CSGALNACT2    55454 ENSG00000169826 10q11.21
## 6    33323_r_at        SFN     2810 ENSG00000175793  1p36.11
## 7     244311_at       <NA>     <NA>            <NA>     <NA>
## 8     220932_at       <NA>     <NA>            <NA>     <NA>
## 9     205508_at      SCN1B     6324 ENSG00000105711  19q13.1
## 10    209596_at      MXRA5    25878 ENSG00000101825  Xp22.33
## 11    215180_at       <NA>     <NA>            <NA>     <NA>
## 12 1560638_a_at       <NA>     <NA>            <NA>     <NA>
## 13  201852_x_at     COL3A1     1281 ENSG00000168542     2q31
## 14    229947_at       PI15    51050 ENSG00000137558  8q21.11
## 15  221731_x_at       VCAN     1462 ENSG00000038427   5q14.3
```

We may exploit the output of the *annotate* function to extent our analysis. For instance, we may perform *pathway analysis* on the exemplars. For this purpose we will utilize the *probes2pathways* function that utilizes the *reactome.db* package.This function employs the probe ids to identify the relevant pathways.

```
probes2pathways(gene.info)

##                                                  1474244
##        "Homo sapiens: Extracellular matrix organization"
```

```
##                                                    1566948
##                  "Homo sapiens: Elastic fibre formation"
##                                                    2129379
## "Homo sapiens: Molecules associated with elastic fibres"
##                                                    1474228
##  "Homo sapiens: Degradation of the extracellular matrix"
```

3.2 Network characteristics

Regarding the network charcteristics, we compute through the *netwAttr* function three different types of centralities (degree, closeness, betweenness) and a meassure for clustering coefficient called transitivity. The degree centrality of a node refer to the number of connections or edges of that node to other nodes.The closeness centrality describes the reciprocal accumulated shortest length distance from a node to all other connected nodes.The betweeness centrality depicts the number of times a node intervenes along the shortest path of two other nodes. Transitivity meassures the degree of nodes to create clusters within a network.For all four network meassures we provide both global and local values. Furthermore, we compose an edge list (Node1-Node2-weight) based on the *N* top ranked genes. We may exploit that meassures to depict the exemplars' network characteristics

```
net.attr <- netwAttr(out.clL2)
wDegreeL <- net.attr@degree$WdegreeL[out.clL2@exemplars]
wClosenessL <- net.attr@closeness$WclosenessL[out.clL2@exemplars]
wBetweenessL <- net.attr@betweenness$WbetweennessL[out.clL2@exemplars]
wTransitivityL <- net.attr@transitivity$WtransitivityL[out.clL2@exemplars]
```

```
Global.val <- c(net.attr@degree$WdegreeG, net.attr@closeness$WclosenessG,
    net.attr@betweenness$WbetweennessG, net.attr@transitivity$WtransitivityG)
```

```
Global.val <- round(Global.val, 2)
exempl.netattr <- rbind(wDegreeL, wClosenessL, wBetweenessL, wTransitivityL)
```

```
netAttr <- cbind(Global.val, exempl.netattr)
netAttr <- t(netAttr)
netAttr
```

```
##                wDegreeL wClosenessL wBetweenessL wTransitivityL
## Global.val       330.18        0.93       741.81           0.57
## 215717_s_at      308.35        1.25       886.00           0.14
## 1561358_at       346.92        1.34      1141.00           0.14
## 222752_s_at      327.89        0.65         0.00           0.14
## 233922_at        317.58        0.79         2.00           0.15
```

```
## 218871_x_at    293.73         0.53        768.00            0.14
## 33323_r_at     338.19         0.27          0.00            0.13
## 244311_at      294.80         0.63          0.00            0.15
## 220932_at      359.10         0.66          0.00            0.14
## 205508_at      309.07         0.89          4.00            0.14
## 209596_at      345.13         1.34        278.00            0.14
## 215180_at      333.37         1.37       1440.00            0.14
## 1560638_a_at   368.23         1.38       4615.00            0.14
## 201852_x_at    353.34         0.93         24.67            0.15
## 229947_at      317.11         1.19        496.00            0.15
## 221731_x_at    331.01         0.61         14.00            0.15
```

and identify potential hubs.The calculations of this example are based on the "clr" network reconstruction method. However, there are for the time being two more options, including the "aracne.a" and "aracne.m".

```
# For local degree > global + standard deviation
sdev <- sd(net.attr@degree$WdegreeL)
msd <- net.attr@degree$WdegreeG + sdev
hubs <- wDegreeL[which(wDegreeL > msd)]
hubs

##    220932_at 1560638_a_at
##       359.10       368.23
```

Finally, we may plot the network for those nodes that their local weighted degree is greater than Global weithed degree plus 2 times the standard deviation. We set this rule for both significance and illustartion purposes (that edge list has dimension 604 x 3).

```
sdev <- sd(net.attr@degree$WdegreeL)
ms2d <- net.attr@degree$WdegreeG + 2 * sdev
net <- net.attr@degree$WdegreeL[which(net.attr@degree$WdegreeL >
    ms2d)]
idx <- which(net.attr@edgelist[, 1] %in% names(net))
new.edgeList <- net.attr@edgelist[idx, ]
dim(new.edgeList)

## [1] 604   3

require(igraph)
g = graph.data.frame(new.edgeList, directed = FALSE)
# tkplot(g,layout=layout.fruchterman.reingold)
```

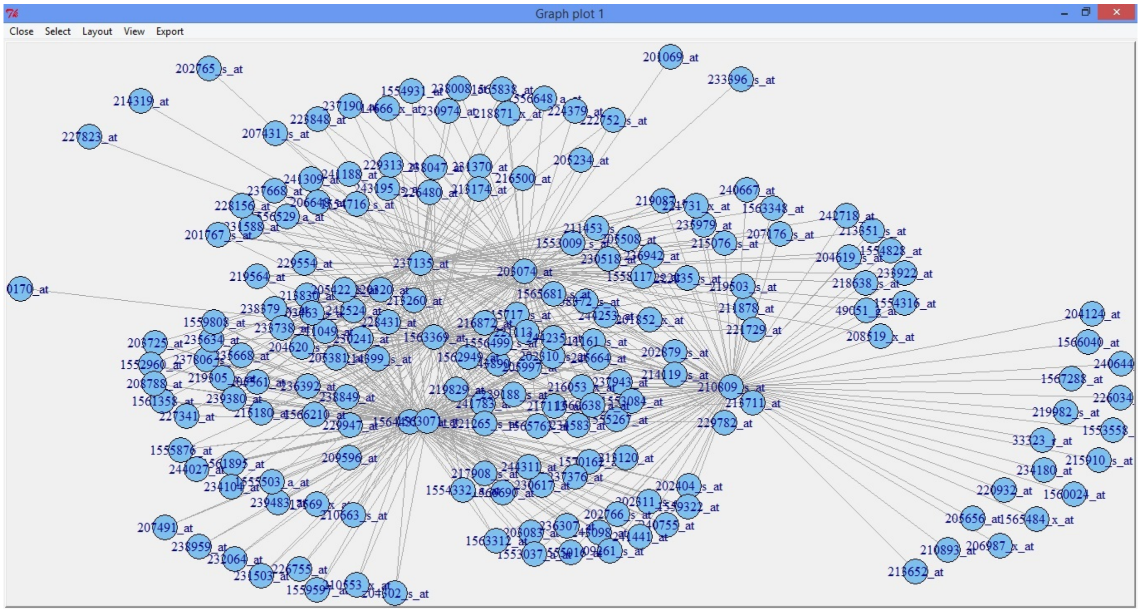

Figure 2: Degree centrality network

4 Reporting

The overall analysis is summarized in an **html** report produced by the *report* function. It covers the dataset repsresentation depicting the samples' names and their respective class labels, the exemplars section where statistical results and network characteristcs are included. The classification performance section illustrates the performance metrics achieved in either cross-validation or hold-out validation.Finally, several annotation info are presented if an annotation analysis has occured.

5 Session info

```
sessionInfo()

## R Under development (unstable) (2014-10-29 r66891)
## Platform: x86_64-w64-mingw32/x64 (64-bit)
##
## locale:
## [1] LC_COLLATE=English_United States.1252  LC_CTYPE=English_United States.1252
## [3] LC_MONETARY=English_United States.1252 LC_NUMERIC=C
## [5] LC_TIME=English_United States.1252
##
## attached base packages:
## [1] stats4    parallel  stats     graphics  grDevices utils     datasets  methods
```

```
## [9] base
##
## other attached packages:
##  [1] igraph_0.7.1         reactome.db_1.48.0   hgu133plus2.db_3.0.0 org.Hs.eg.db_3.0.0
##  [5] AnnotationDbi_1.30.1 GenomeInfoDb_1.4.0   IRanges_2.2.1        S4Vectors_0.6.0
##  [9] mAPKLData_0.99.1     mAPKL_1.0.1          RSQLite_1.0.0        DBI_0.3.1
## [13] Biobase_2.28.0       BiocGenerics_0.14.0  knitr_1.10.5
##
## loaded via a namespace (and not attached):
##  [1] ade4_1.7-2         apcluster_1.4.1    BiocStyle_1.6.0    class_7.3-12
##  [5] cluster_2.0.1      clusterSim_0.44-2 e1071_1.6-4        evaluate_0.7
##  [9] formatR_1.2        grid_3.2.0         highr_0.5          lattice_0.20-31
## [13] limma_3.24.3       magrittr_1.5       MASS_7.3-40        Matrix_1.2-0
## [17] multtest_2.24.0    parmigene_1.0.2    R2HTML_2.3.1       Rcpp_0.11.6
## [21] rgl_0.95.1247      splines_3.2.0      stringi_0.4-1      stringr_1.0.0
## [25] survival_2.38-1    tools_3.2.0
```

6 Reference

Sakellariou, A., D. Sanoudou, and G. Spyrou. "Combining Multiple Hypothesis Testing and Affinity Propagation Clustering Leads to Accurate, Robust and Sample Size Independent Classification on Gene Expression Data. " BMC Bioinformatics 13 (2012): 270.

http://www.ncbi.nlm.nih.gov/geo/query/acc.cgi?acc=GSE5764

mAP-*KL* Analysis Report

Sat Dec 06 20:54:13 2014

Data Samples

Training Set	Class Labels	Validation Set	Class Labels
GSM134584	0	GSM134691	0
GSM134690	0	GSM134588	0
GSM134693	0	GSM134688	0
GSM134696	0	GSM134694	0
GSM134586	0	GSM134697	0
GSM134589	0	GSM134700	0
GSM134699	0	GSM134687	0
GSM134702	0	GSM134709	0
GSM134705	0	GSM134710	1
GSM134708	0	GSM134698	1
GSM134703	0	GSM134689	1
GSM134706	0	GSM134692	1
GSM134701	1		
GSM134704	1		
GSM134587	1		
GSM134591	1		
GSM134707	1		
GSM134695	1		

Exemplars

Exemplars	Adj.p-value	p-value	FC	wt.degree	wt.closeness	wt.betweenness	wt.transitivity
215717_s_at	0.885	0.001	2.252	308.35	1.25	886	0.14
1561358_at	0.928	0.001	1.246	346.92	1.34	1141	0.14
222752_s_at	0.947	0.001	0.88	327.89	0.65	0	0.14
233922_at	0.952	0.001	-1.102	317.58	0.79	2	0.15
218871_x_at	0.987	0.001	0.901	293.73	0.53	768	0.14
33323_r_at	0.997	0.001	-0.701	338.19	0.27	0	0.13
244311_at	0.999	0.003	-1.355	294.8	0.63	0	0.15
220932_at	0.999	0.002	-1.069	359.1	0.66	0	0.14
205508_at	1	0.001	1.349	309.07	0.89	4	0.14
209596_at	1	0.002	1.662	345.13	1.34	278	0.14
215180_at	1	0.003	-1.888	333.37	1.37	1440	0.14
1560638_a_at	1	0.001	-2.336	368.23	1.38	4615	0.14
201852_x_at	1	0.002	2.239	353.34	0.93	24.67	0.15
229947_at	1	0.003	-4.004	317.11	1.19	496	0.15
221731_x_at	1	0.001	2.046	331.01	0.61	14	0.15

Classification Performance

(Hold-out Validation)

AUC	Accuracy	MCC	Specificity	Sensitivity
0.94	92.00	0.84	0.88	1.00

Genome Annotation

PROBE ID	SYMBOL	ENTREZ ID	ENSEMBL ID	MAP
215717_s_at	FBN2	2201	ENSG00000138829	5q23-q31
1561358_at	TXLNA	200081	ENSG00000084652	1p35.1
222752_s_at	TMEM206	55248	ENSG00000065600	1q32.3
233922_at	NA	NA	NA	NA
218871_x_at	CSGALNACT2	55454	ENSG00000169826	10q11.21
33323_r_at	SFN	2810	ENSG00000175793	1p36.11
244311_at	NA	NA	NA	NA
220932_at	NA	NA	NA	NA
205508_at	SCN1B	6324	ENSG00000105711	19q13.1
209596_at	MXRA5	25878	ENSG00000101825	Xp22.33
215180_at	NA	NA	NA	NA
1560638_a_at	NA	NA	NA	NA
201852_x_at	COL3A1	1281	ENSG00000168542	2q31
229947_at	PI15	51050	ENSG00000137558	8q21.11
221731_x_at	VCAN	1462	ENSG00000038427	5q14.3

Figure 3: mAPKL analysis report
