# Supplementary figures and images for: mAPKL: R/ Bioconductor package for detecting gene exemplars and revealing their characteristics
Source: BMC Bioinformatics. 2015 Sep 15;16(1):291. doi: 10.1186/s12859-015-0719-5 (PMC4572678; doi:10.1186/s12859-015-0719-5)

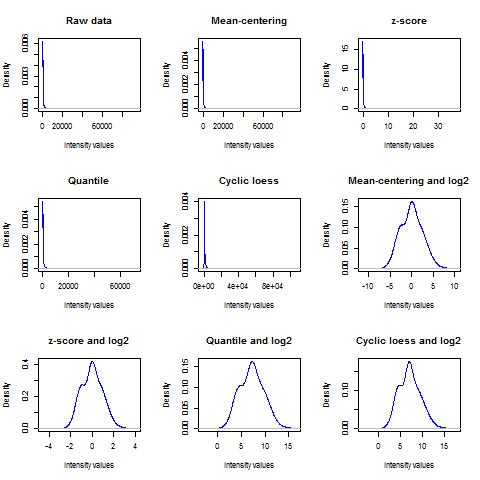

Supplement: Additional file 1: — Package ‘mAPKL_1.1.1.tar.gz’ including source code, documentation. (GZ 1979 kb) [file 12859_2015_719_MOESM1_ESM.gz › mAPKL/man/figures/density_train.jpeg]

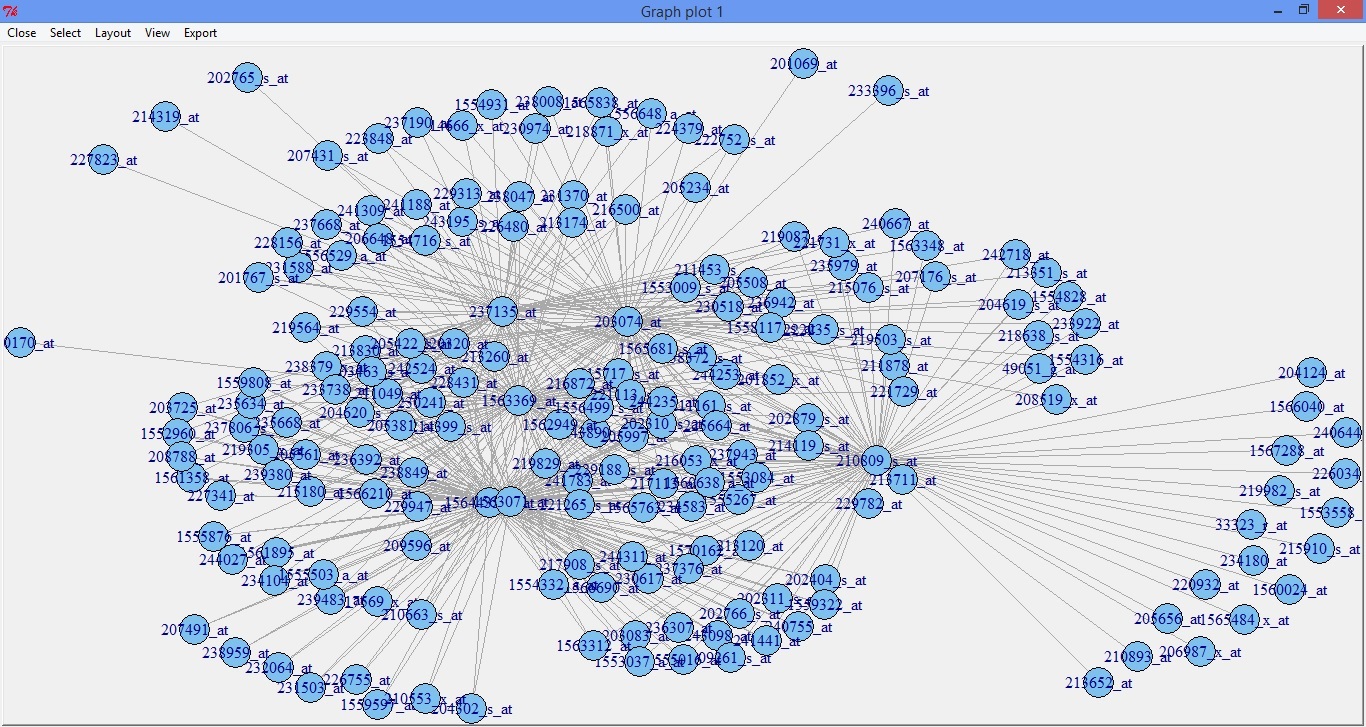

Supplement: Additional file 1: — Package ‘mAPKL_1.1.1.tar.gz’ including source code, documentation. (GZ 1979 kb) [file 12859_2015_719_MOESM1_ESM.gz › mAPKL/man/figures/network.jpg]

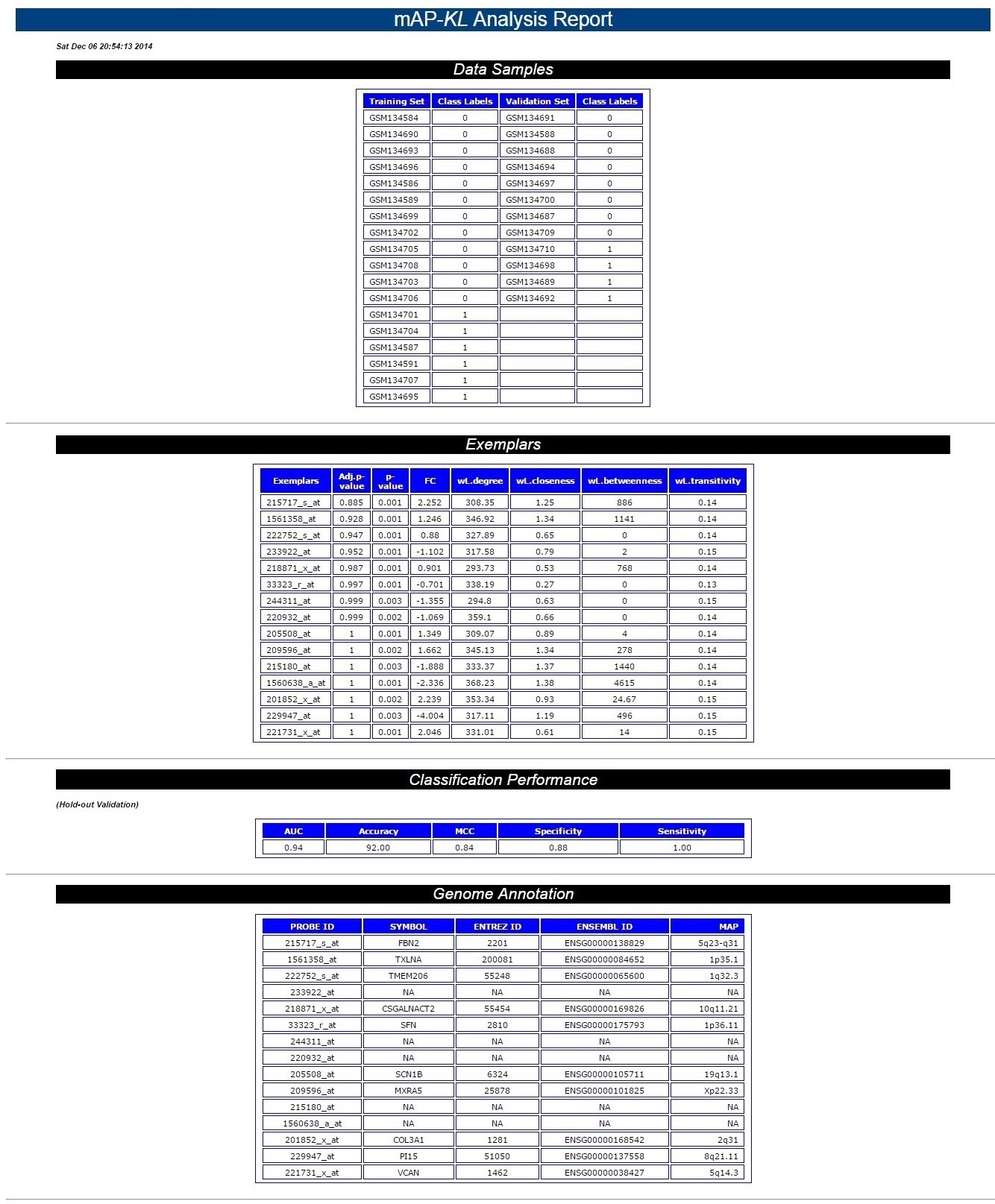

Supplement: Additional file 1: — Package ‘mAPKL_1.1.1.tar.gz’ including source code, documentation. (GZ 1979 kb) [file 12859_2015_719_MOESM1_ESM.gz › mAPKL/man/figures/report.jpg]
